# *Cuscutae Semen* in depression-induced ovarian dysfunction: metabolomics with UPLC-QToF-MS in female mice

**DOI:** 10.3389/fmolb.2025.1595602

**Published:** 2025-04-30

**Authors:** Ying Xie, Zhaoxiang Zeng, Jinrong Zhang, Qiangqiang Han, Chengwu Song, Shuna Jin, Min Zhao

**Affiliations:** ^1^ School of Basic Medical Sciences, Hubei University of Chinese Medicine, Wuhan, Hubei, China; ^2^ School of Pharmacy, Hubei University of Chinese Medicine, Wuhan, Hubei, China; ^3^ SpecAlly Life Technology Co., Ltd., Wuhan, Hubei, China; ^4^ Hubei Shizhen Laboratory, Wuhan, Hubei, China

**Keywords:** *Cuscutae Semen*, depression, ovarian dysfunction, metabolomics, ultra-performance liquid chromatography coupled with quadrupole time-of-flight mass spectrometry

## Abstract

The increasing prevalence of depression profoundly affects female ovarian health. Although *Cuscutae Semen* (CS) is acknowledged for treating reproductive disorders, its pharmacological mechanisms in depression-induced ovarian dysfunction remain insufficiently explored. This study investigated CS’s effects in a chronic unpredictable mild stress (CUMS) mouse model of depression. Mice were divided into control, CUMS model, CS treatment and estradiol treatment group. Behavioral and biochemical analyses assessed depressive-like behaviors and hormone levels. Untargeted metabolomics utilizing ultra-performance liquid chromatography coupled with quadrupole time-of-flight mass spectrometry was applied to identify differential metabolites of CS in the treatment of depression-induced ovarian dysfunction. These findings were confirmed through real-time quantitative polymerase chain reaction assays. Based on the outcomes from behavioral and biochemical assays, CS effectively ameliorated the chronic unpredictable mild stress-induced reproductive ailment in mice. Ten differential metabolites were identified, highlighting the impact of CUMS and CS’s ameliorative effects. Pathways linked to arachidonic acid metabolism, glycerophospholipid metabolism, linoleic acid metabolism, and steroid hormone biosynthesis were involved. Seven target genes further validated the metabolomic analysis. This study provides strong evidence of CS’s therapeutic potential in alleviating depression-induced ovarian dysfunction, shedding light on its pharmacological mechanisms and supporting its use as a functional medical food.

## 1 Introduction

Depression is a prevalent mental health problem, often characterized by anhedonia, feelings of worthlessness, impaired concentration, disrupted sleep, and suicidal ideation (Misztak et al., 2021). It has an early onset, high recurrence rate, and often becomes chronic, significantly impacting individuals’ quality of life (Richards, 2011). Globally, approximately 3.8% of the population is affected by depression, with women being nearly twice as likely to suffer from the condition as men (Kuehner, 2017; Koric et al., 2021). In addition to its psychological toll, depression has a profound impact on female reproductive health, leading to complications such as menstrual irregularities and impaired ovarian function (Koric et al., 2021; Luo et al., 2021). Due to the heightened recurrence rate of depression and the difficulty of recovery from reproductive diseases, long-term use of conventional pharmaceutical therapy may result in increased adverse reactions, while short-term use may lack therapeutic efficacy (Zeng et al., 2022).

Given these challenges, there is a growing need for alternative therapies that address both the psychiatric and reproductive components of depression. Traditional Chinese medicine (TCM) has gained attention for its potential to manage chronic diseases with fewer side effects (Xiang et al., 2020). *Cuscutae Semen* (CS), a well-known TCM derived from the seeds of *Cuscuta chinensis* Lam., is rich in bioactive compounds such as flavonoids, polysaccharides, and alkaloids (Yang et al., 2016; Yang et al., 2017). CS has demonstrated therapeutic effects in both depressive disorders and female reproductive dysfunctions, including polycystic ovary syndrome and premature ovarian failure (Miao et al., 2019; Han et al., 2020). However, it is unclear whether and how CS can effectively regulate depression-induced reproductive ailments, which potentially impedes its sustained utilization.

To explore these mechanisms, metabolomics offers a powerful, high-throughput approach to identify biomarkers and metabolic pathways involved in disease and treatment (Cui et al., 2018). Untargeted metabolomics using liquid chromatography-mass spectrometry (LC-MS) is particularly effective in uncovering complex biological processes (Zeng et al., 2021). Given the intricate and not entirely understood etiopathogenesis of depression, employing an appropriate methodology to explore its treatment mechanisms is imperative (Orsolini et al., 2022). Consequently, untargeted LC-MS-based metabolomics was used to investigate the regulatory effect of CS in the treatment of depression-induced reproductive functional maladies.

In this study, the depression-induced ovarian dysfunction was constructed by chronic unpredictable mild stress (CUMS). Animal experiments were devised to scrutinize the impairment of reproductive function induced by CUMS and the therapeutic efficacy of CS in female mice. Then, employing non-targeted ultra-performance liquid chromatography coupled with quadrupole time-of-flight mass spectrometry (UPLC-QToF-MS) metabolomics, we aim to elucidate potential biomarkers and metabolic pathways associated with the therapeutic efficacy of CS. Finally, the potential regulatory mechanisms underlying the treatment of ovarian dysfunction with CS were further elucidated using real-time quantitative polymerase chain reaction (RT-qPCR) methods.

## 2 Materials and methods

### 2.1 Materials and reagents

Estradiol valerate was obtained from Bayer Healthcare Co., Ltd., Guangzhou Branch, with the approval number of J20171038. HPLC-grade methanol was acquired from Fisher Scientific (Fair Lawn, NJ, United States). Enzyme-linked immunosorbent assay (ELISA) kits for measuring anti-mullerian hormone (AMH) (Lot No. MM-44204M2), luteinizing hormone (LH) (Lot No. MM-44039M2), estradiol (E2) (Lot No. MM-0566M1) and follicle-stimulating hormone (FSH) (Lot No. MM-45654M2) in mice were procured from Wuhan Enzyme Free Biotechnology Co., Ltd. An RNA extraction kit was purchased from TaKaRa, Dalian, China. Deionized water was prepared using a Milli-Q water system (Millipore, Bedford, MA, United States).

### 2.2 Preparation of CS extract

CS (batch No. 210801) was collected from Nanjing Shangyuantang Pharmaceutical Joint Stock Co., Ltd. The identification and authentication were undertaken by Professor Xiaoming Yu at Hubei University of Chinese Medicine. CS was washed to eliminate impurities and subsequently soaked in water for 4 h. Subsequently, gently simmer CS for 30 min, then filter the solution. Repeat this procedure twice, combine the filtrates, and adjust the concentration to 0.5 g/mL before storing the solution in a refrigerator at 4°C for preservation.

### 2.3 Animals and treatment

Seven-week-old female C57BL/6 mice (SPF grade) were provided by Liaoning Biotechnology Co., Ltd., with certification No. SCXK (Liao) 2020-0001. The mice were given standard food and drink freely, adhering to a 12-h light-dark cycle. After a 7-day adaptation period, the mice were randomly allocated into four groups: the control (CON) group, CUMS group, CS treatment (CUMS+CS), and estradiol treatment (CUMS+E) group, with six in each group, as described in Table 1. The construction method for the CUMS model was primarily based on previously published papers with slight modifications (Jiang et al., 2018; Song and Wang, 2021; Li et al., 2022), and mice were randomly exposed to a series of twelve mild stressors, including bondage 4 h, wet sawdust 12 h, food-depriving 12 h, reversed light/dark cycle, water-depriving 12 h, tail suspension 2 h, crowded housing 5 h, empty cage 12 h, cold water swimming 5 min, noise interference 15 min, cage tilt 12 h, tail clamp 1 min, as detailed in Supplementary Table S1. Observations of the mice were made, including the weekly measurement of their body weights. Behavioral assessments commenced after the stressor modeling. Throughout the model development phase, CS was delivered via intragastric gavage at a dose of 2.6 g kg^−1^ per day, with daily administrations before modeling for a total of 28 days. In the E2 treatment group, the tablets were ground into a powder, dissolved in normal saline, and administered at a dose of 0.13 mg kg^−1^ per day for a total of 28 days. CON and CUMS groups were treated with normal saline, using the same average solution volume as that administered to the treatment group. All mice were sacrificed by cervical dislocation following blood collection under pentobarbital anesthesia. Animal experiments were approved by the Institutional Animal Care and Use Committee of Hubei University of Chinese Medicine (Approval No. HUCMS202209003), and the approval document was found in the Supplementary Figure S1.

**TABLE 1:** Timeline of each group during the modeling phase and treatment phase.

No.	Groups	Duration	
		1–4 weeks	5–8 weeks
1	CON	None	None
2	CUMS	Chronic unpredictable mild stress modeling	None
3	CUMS+CS	Chronic unpredictable mild stress modeling	*Cuscutae Semen* treatment
4	CUMS+E	Chronic unpredictable mild stress modeling	Estradiol treatment

“CON”, “CUMS”, “CUMS+CS” and “CUMS+E” represented control group, chronic unpredictable mild stress group, *Cuscutae Semen* treatment group and estradiol treatment group, respectively.

### 2.4 Behavioral test

#### 2.4.1 Sucrose preference test

The sucrose preference test (SPT) in mice was performed following a previous method with minor modifications (Forbes et al., 1996). During the initial phase of adaptation, each mouse was housed in a single cage and presented with two bottles of a 1% sucrose aqueous solution. The next day, regular drinking water replaced one of the bottles containing the sucrose solution, and the positions of the bottles were changed periodically. At the end of the adaptation period, all mice underwent a 24-h fast before the experiment. In the test, mice were given the choice between a 1% sucrose aqueous solution and regular drinking water. The following day, the preference of mice for sucrose water was determined based on the difference in the amount of water consumed before and after the experiment.

#### 2.4.2 Tail suspension test

The tail suspension test (TST) in mice was slightly adapted according to the previous description (Basu Mallik et al., 2022). The testing device was a rectangular box with a hook fixed with tape to hang the tail of the mice upside down. During the entirety of the experiment, the tail tip of the mouse was approximately 20 cm away from the bottom of the enclosure. The mice were suspended for a duration of 6 min, and the duration time of immobility was recorded when the mice were passively suspended and completely immobile in the last 4 min.

#### 2.4.3 Focused swimming test

The focused swimming test (FST) in mice was subtly modified in accordance with the prior description (Porsolt et al., 1977). The experimental device was a transparent cylindrical vessel filled with tap water (25°C), and the water level was controlled within the range where the hind limbs of mice cannot touch the bottom. In the experimental setting, each mouse underwent an individualized swimming session lasting 5 min, during which the immobility period in the last 4 min (moving the head without exposing to water) was recorded. Then, the mice were taken out and carefully dried to restore their body temperature. After each mouse was taken out, the container was cleaned and the water was replenished.

### 2.5 Histological evaluation

The ovarian tissues obtained from the experiment were placed in a −80°C freezer and used for the subsequent histological evaluation. Ovarian tissue was subjected to fixation in 4% paraformaldehyde, followed by alcohol gradient dehydration, xylene cleaning, and paraffin embedding. After deparaffinization, the tissue was stained with hematoxylin and eosin, and microscopic examination was conducted.

### 2.6 Biochemical evaluation

Blood samples were collected from each mouse by eyeball sampling. The samples obtained from the experiment underwent centrifugation at 4,000 revolutions per minute for 10 min. The supernatant was placed in a −80°C freezer and used for subsequent biochemical evaluation. Following the protocol provided by the manufacturer, the ELISA technique was used to measure levels of AMH, LH, E2 and FSH.

### 2.7 Sample preparation

The ovarian tissues from the experiment, previously stored at −80°C, were utilized for metabolomic sample preparation. Mixing 5 mg of sample with 150 μL of a methanol solution containing 100 ng/mL of curcumin (used as an internal standard), and incorporating the blend with two zirconium balls to form a homogenate. The mixture underwent centrifugation at 12,000 rpm for 10 min at 4°C. An equivalent volume of the supernatant was transferred into sample bottles. Aliquots from all supernatant samples were combined to create a unified sample for quality control (QC).

### 2.8 Untargeted UPLC-QToF-MS-based metabolomics

Utilizing the Acquity UPLC H-Class system (Waters Corporation, Milford, MA, United States), equipped with a Waters Acquity UPLC BEH C18 column (2.1 × 100 mm, 1.7 μm), the samples were subjected to analysis. A solution comprised of water and formic acid in a ratio of 1,000:1 served as mobile phase A, while methanol was employed as mobile phase B. The gradient conditions were as follows: 0 min, 10% B; 15.0 min, 95% B; 20.0 min, 95% B; 21.0 min, 10% B; 25.0 min, 10% B. The flow rate was 0.3 mL/min, with the column temperature set at 40°C. Exactly 2.0 μL of the sample was injected.

Utilizing the Waters Xevo G2-XS QToF system, coupled with an electrospray ionization source, the analysis was conducted through mass spectrometry. The acquisition of data was conducted in sensitivity analysis mode, utilizing both positive and negative ion electrospray methods (positive ion mode for metabolomics analysis and negative ion mode for the determination of metabolite structures). The optimum operational parameters were established as follows: desolvation temperature at 500°C, desolvation gas flow at 1,000 L/Hr, cone voltage at 20 V, cone gas flow at 50 L/Hr, source temperature at 100°C, capillary voltage at 3 kV and collision energy at 30–40 eV. The mass ranges were configured for a full scan from *m/z* 100–1,200 Da, with a scan duration of 1.0 s. The MS^E^ mode was used to collect data. The QToF-MS was calibrated with sodium formate in the range of 100–1,200 Da in both ionization modes. Accurate mass measurements were performed by infusing leucine-enkephaline lock mass compound (ESI + *m/z* 556.2771, ESI − *m/z* 554.2615) with alternating between the sample and the lock mass in 45 and 60 s intervals for ESI+ and ESI−, respectively. The lock spray flow rate was 10 μL/min, 0.5 s scan time, cone voltage 30 V, collision energy 4 eV. The data-independent acquisition was performed in continuum mode with MassLynx™ V4.1 workstation (Waters Corporation, MA, United States).

### 2.9 RT-qPCR analysis

The ovarian tissues from the experiment, previously stored at −80°C, were employed for subsequent RNA extraction. RNA was extracted from ovarian tissue using an RNA extraction kit for animals to obtain total RNA. ABScript Neo RT Master Mix for qPCR with gDNA Remover (ABclonal, Wuhan, China) was used by reverse transcription. The gene expression levels in the samples were quantified using 2X Universal SYBR Green Fast qPCR Mix (ABclonal, Wuhan, China) and a RT-qPCR instrument. The gene expression data underwent normalization and computation through the 2^−ΔΔCT^ method. The primer sequences utilized for RT-qPCR were detailed in Supplementary Table S2.

### 2.10 Data analysis

The raw data acquired from UPLC-QToF-MS analysis was gathered using MassLynx™ V4.1. Following that, MarkerLynx XS (Waters Corporation, MA, United States) was employed for peak extraction, alignment, and isotope peak exclusion. Before undertaking untargeted metabolomic profiling, adjustments were made to the raw data table to mitigate the presence of apparent false positives. Adjustments were made in accordance with the following criteria (not necessarily in this order) for methodological construction: (1) retention of peaks between 2 and 20 min; (2) alignment of peaks with a mass tolerance of 0.01 Da; (3) noise elimination threshold set at 80; (4) detection of peaks following the “50% rule” to minimize missing value input.

Multivariate statistical analysis was conducted utilizing SIMCA 14.1 software (Umetrics AB, Umeå, Sweden). Statistical analyses were performed using the Mann-Whitney *U* test in IBM SPSS 26.0 software (SPSS Inc.). Graphs were generated using Graph Pad Prism 7.0 (GraphPad Software Inc., San Diego, CA) and Origin 2021 (OriginLab Corporation, United States). A visual integrated metabolic map of primary metabolic pathways and associated metabolites was constructed based on logarithmic transformation data with a base of 10, guided by the Kyoto Encyclopedia of Genes and Genomes (KEGG). Various software applications are utilized on Windows 10 Professional (Microsoft, Redmond, WA, United States), with Microsoft Office Professional Plus 2019 employed for data collation and processing.

## 3 Results

### 3.1 Evaluation of the therapeutic effect against CUMS-induced model

**FIGURE 1:**
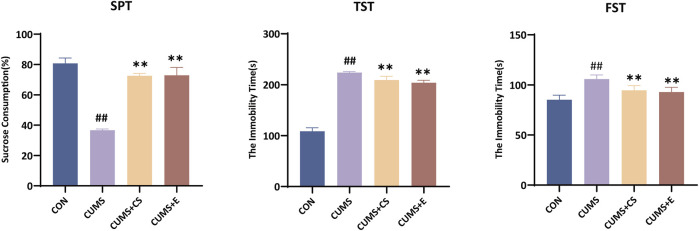
Evaluation of sucrose preference test (SPT), tail suspension test (TST) and focused swimming test (FST) for measuring depressive-like behavior. “CON”, “CUMS”, “CUMS+CS” and “CUMS+E” represented control group, chronic unpredictable mild stress group, *Cuscutae Semen* treatment group and estradiol treatment group, respectively. Mann-Whitney *U* test was used to calculate significant difference. ^##^ *P* < 0.01, compared with CON group. ^**^ *P* < 0.01, compared with CUMS group. Each group contained six samples, maintaining consistency across all groups.

To ascertain the success of the modeling process and the potential alleviative effects of CS on depression, behavioral tests were conducted. As illustrated in Figure 1, the results on the therapeutic effect of CS against CUMS-induced model were constructing by behavioral indicators of mice. In the SPT, sugar water preference of mice was decreased significantly after modeling (*P* < 0.01), while it was increased after administration of CS and E2 (*P* < 0.01). Compared with the CON group, the duration of immobility in the CUMS group was significantly longer in the TST (*P* < 0.01). Following the treatment, the immobility time of the mice demonstrated a significant decrease in the CUMS+CS group (*P* < 0.01) and the E2 group (*P* < 0.01) when compared with the CUMS group. In the FST, the immobility time of the mice in the CUMS group was significantly increased compared to the CON group (*P* < 0.01). Compared to the CUMS group, the immobility time displayed a downregulated pattern in administration group of CS (*P* < 0.01) and E2 (*P* < 0.01).

### 3.2 Pathological indicators of the therapeutic effect

**FIGURE 2:**
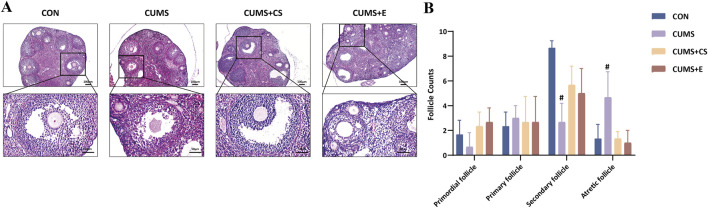
Ovarian pathological examinations **(A)** and enumeration of follicles at diverse development stages **(B)** in female mice.“CON”, “CUMS”, “CUMS+CS” and “CUMS+E” represented control group, chronic unpredictable mild stress group, *Cuscutae Semen* treatment group and estradiol treatment group, respectively. Mann-Whitney *U* test was used to calculate significant difference. ^#^ *P* < 0.01, compared with CON group. Each group contained six samples, maintaining consistency across all groups.

To investigate the reproductive functionality of female mice, we conducted ovarian pathological examinations. Figure 2 depicted histopathological sections and follicle count of mice after the modeling and healing. In the CON group, ovarian follicles were well-developed condition across various stages. After modeling, mice in the CUMS group exhibited poor follicular development at all stages, connective tissue hyperplasia in the medullary portion and capillary rupture with hemorrhage in the ovary. Moreover, the number of secondary follicles showed a significant decrease (*P* < 0.05). Furthermore, compared to the CON group, the number of ovarian follicles decreased at various stages in the CUMS group, but significantly increased during the atretic state (*P* < 0.05). Following administration of CS and E2, ovarian development in mice improved significantly, and the number of follicles at various stages increased.

### 3.3 Biochemical indicators of the therapeutic effect

**FIGURE 3:**
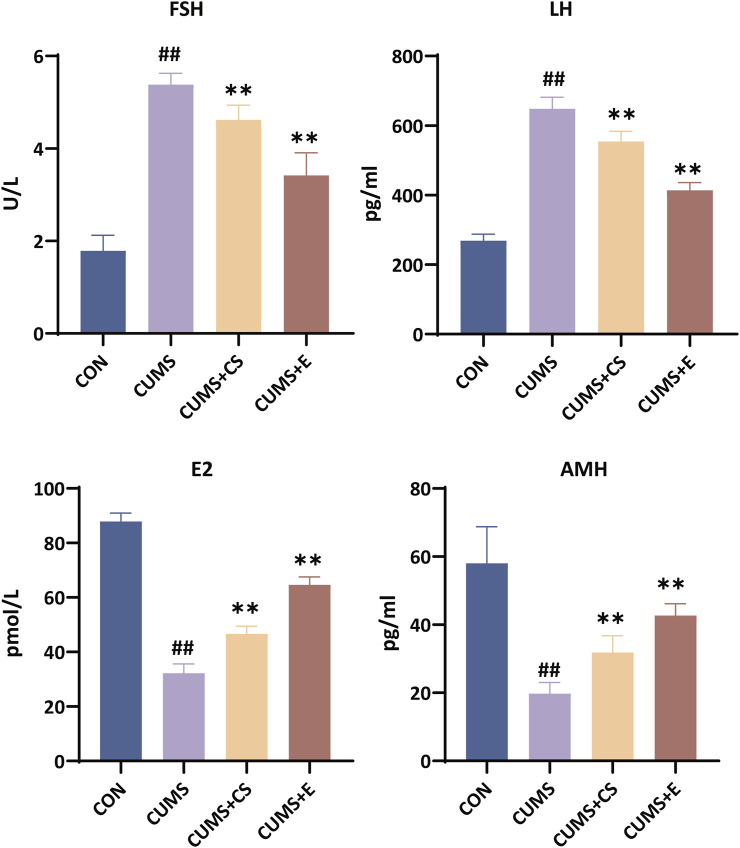
Assessment of follicle-stimulating hormone (FSH), luteinizing hormone (LH), estradiol (E2) and anti-mullerian hormone (AMH) indicators in female mice. “CON”, “CUMS”, “CUMS+CS” and “CUMS+E” represented control group, chronic unpredictable mild stress group, *Cuscutae Semen* treatment group and estradiol treatment group, respectively. Mann-Whitney *U* test was used to calculate significant difference. ^##^ *P* < 0.01, compared with CON group. ^**^ *P* < 0.01, compared with CUMS group. Each group contained six samples, maintaining consistency across all groups.

As depicted in Figure 3, serum FSH levels in the CUMS group were significantly increased (*P* < 0.01), whereas they were significantly reduced after administration of the CUMS+CS (*P* < 0.01) and CUMS+E groups (*P* < 0.01). The content of LH in the serum of mice after modeling exhibited a significant upward pattern (*P* < 0.01), but it showed an opposite trend following administration (*P* < 0.01). Moreover, the level of E2 in the serum of the modeling group was decreased significantly (*P* < 0.01), and it increased significantly after the treatment of CS (*P* < 0.01) and E2 (*P* < 0.01). In comparison to the CON group, the CUMS group exhibited a significant reduction in AMH content (*P* < 0.01), while both the CUMS+CS group (*P* < 0.01) and the CUMS+E group (*P* < 0.01) demonstrated a substantial increase in AMH levels.

### 3.4 Untargeted metabolomic analysis

A total of 8,032 features within the dataset were initially acquired by Markerlynx XS. Following the elimination of false positives, 3,362 features were retained for the metabolomic analysis.

**FIGURE 4:**
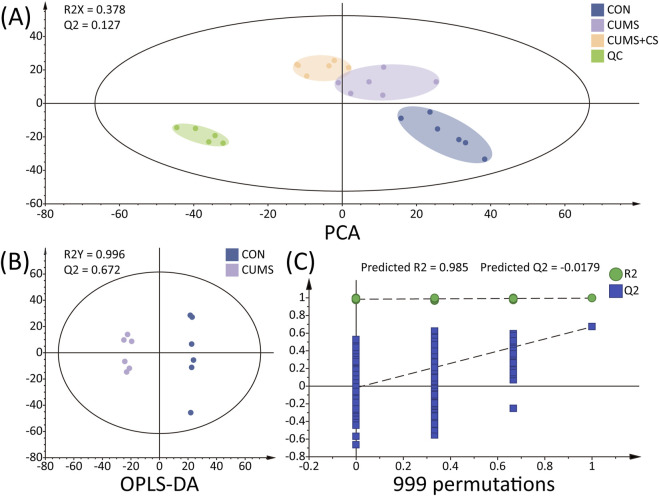
**(A)** Principal component analysis (PCA) of peak areas detected in quality control (QC), control (CON), chronic unpredictable mild stress (CUMS) and *Cuscutae Semen* treatment (CUMS+CS) group. **(B)** Orthogonal partial least squares discrimination analysis (OPLS-DA) score scatter plot in comprising of CON-CUMS. **(C)** Results of 999 permutations for the OPLS-DA model.

As depicted in Figure 4A, the differences in metabolites between CON, CUMS, CUMS+CS, and QC were analyzed using principal component analysis (PCA), with the results illustrated in the score plot. The samples from each group (CON, CUMS and CUMS+CS group) exhibited a conspicuous clustering pattern as well as distinct separation among the groups, indicating that there were significant differences in metabolic profiles, the stability of the modeling method and administration approach. In the PCA score plot, the QC samples demonstrated a tight cluster, indicating the robust stability and reproducibility of the LC-MS measurements. In Figure 4B, the score scatter plots of orthogonal partial least squares discrimination analysis (OPLS-DA) indicated that CON and CUMS group were segregated mutually. The values of R2Y and Q2 were 0.996 and 0.672 for CON and CUMS group, signifying the excellent fitting and prediction reliability. Additionally, the effectiveness and predictive capability of the OPLS-DA model were evaluated through a 999-times permutation test (see Figure 4C). In the permutation plots, each blue Q2 value on the left side was inferior to the original points on the right, and the blue regression line of the Q2 intersected the vertical axis below zero, indicating that the OPLS-DA model did not experience overfitting.

In OPLS-DA model comparing the CON and CUMS groups, 829 features with the variable importance in the projection (VIP) scores >1.2 were retained as important variables. Subsequently, 395 variable features were confirmed after examining precursor ions in both positive and negative modes. After corroborating the mass spectrometry information of compounds, encompassing precursor and daughter ions, through scrutiny against the HMDB database, a selection of 161 pertinent metabolites was retained. In the subsequent step, through Mann-Whitney *U* test (*P* < 0.05), 45 differential metabolites were discerned in comparisons of CON-CUMS. Eventually, 10 differential metabolites with reversal trends after CS administration, compared with the CUMS group, were characterized and preserved. Table 2 presents qualitative and quantitative information on differential metabolites, encompassing metabolite identification number, retention time, mass spectrometry data, mass error, adduct type, fragmentation information, molecular formula, preliminary identification, VIP, and fold change for these metabolites.

**TABLE 2:** Qualitative and quantitative information on differential metabolites.

No.	RT (min.)	Mass (*m/z*)	Mass error (ppm)	Adduct	MS2 fragments	Formula	Tentative identification	VIP	Fold change	
									CUMS/CON	CUMS+CS/CUMS
1	13.20	315.2349	7.93	[M+H]^+^	109.0654 (0.92), 119.0858 (−2.52), 123.0802 (−6.5), 129.0719 (11.62), 131.0860 (−0.76), 143.0863 (1.40), 145.1005 (−8.27), 155.0868 (4.51)	C_21_H_30_O_2_	Progesterone	1.72	0.24	2.98
2	13.91	337.2376	−0.89	[M+H]^+^	119.0858 (2.52), 131.0860 (3.81), 133.1015 (2.25), 145.1030 (12.41), 159.1186 (11.31)	C_20_H_32_O_4_	5-HPETE	1.73	2.89	0.93
3	14.91	319.2257	−5.01	[M+H]^+^	117.0691 (−6.83), 131.0860 (3.81), 133.1002 (−7.51)	C_20_H_30_O_3_	Leukotriene A4	1.52	1.93	0.61
4	15.21	321.2397	−10.27	[M+H]^+^	105.0701 (1.90), 119.0858 (2.52), 121.0995 (−14.04), 131.0860 (3.81), 133.1015 (2.25)	C_20_H_32_O_3_	11,12-Epoxyeicosatrienoic acid	1.31	4.52	0.66
5	15.27	542.3293	8.48	[M+H]^+^	125.0014 (12.80), 184.0736 (1.63), 313.2761 (7.66)	C_28_H_48_NO_7_P	LysoPC(20:5)	1.23	3.77	0.75
6	17.31	301.2108	−11.62	[M+Na]^+^	107.0870 (8.40), 121.1018 (0.83), 123.1165 (−7.31), 135.1181 (5.18), 149.1336 (4.02), 161.1331 (0.62)	C_18_H_30_O_2_	gamma-Linolenic acid	1.72	1.86	0.68
7	17.43	327.2271	−8.86	[M+Na]^+^	121.1018 (0.83), 147.1163 (−7.48), 161.1331 (0.62), 175.1478 (−5.14), 203.1794 (−2.95), 215.1790 (−4.65), 245.2288 (7.75)	C_20_H_32_O_2_	Arachidonic acid	1.72	1.49	0.74
8	17.46	560.3708	2.86	[M+Na]^+^	282.2801 (1.42), 313.2761 (7.66)	C_27_H_56_NO_7_P	LysoPE (22:0)	1.68	2.08	0.58
9	17.48	343.2613	1.17	[M+Na]^+^	121.1018 (4.95), 123.1165 (−2.44), 149.1311 (−9.39), 161.1331 (3.72), 177.1657 (10.72), 261.2248 (13.40)	C_21_H_36_O_2_	Pregnanediol	1.50	0.69	1.2
10	17.60	341.2439	−4.98	[M+Na]^+^	109.1017 (4.58), 153.1283 (5.88), 159.1160 (−5.03), 161.1331 (3.72), 177.1630 (−4.52)	C_21_H_34_O_2_	Pregnanolone	1.46	0.63	1.08

The values in brackets following the secondary fragments represent the ppm values of both the actual and reference secondary fragments.

**FIGURE 5:**
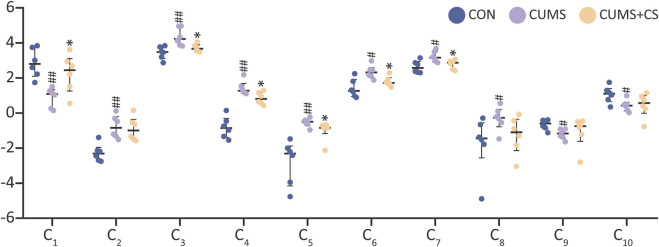
The relative contents of 10 discriminant metabolites in the control (CON), chronic unpredictable mild stress (CUMS), and *Cuscutae Semen* treatment (CUMS+CS) group. The data on the relative contents of differential metabolites were subjected to logarithmic transformation with a base of 2. Mann-Whitney *U* test was used to calculate significant difference. ^#^ *P* < 0.05, ^##^ *P* < 0.01, compared with CON group. ^*^ *P* < 0.05, compared with CUMS group.

In accordance with the quantitative results of differential metabolites, three metabolites in the CUMS group exhibited the downregulated tendency compared with the CON group, whereas seven metabolites displayed the opposite pattern. Interestingly, the metabolites with downward trend were exclusively comprised of pregnane steroids. To understand the alternation profile of metabolites during modeling and administration, the quantitative data was visualized using interleaved scatter pattern in Figure 5. Following the outcomes of Mann-Whitney *U* test (*P* < 0.05), the discrepancy of metabolites between the CON and CUMS groups was even significant. However, six metabolites of the CUMS and CUMS+CS groups manifested significant differences, including progesterone, leukotriene A4, 11,12-epoxyeicosatrienoic acid, LysoPC (20:5), gamma-linolenic acid and arachidonic acid.

### 3.5 Metabolic pathways of CS against CUMS-induced ovarian dysfunction

**FIGURE 6:**
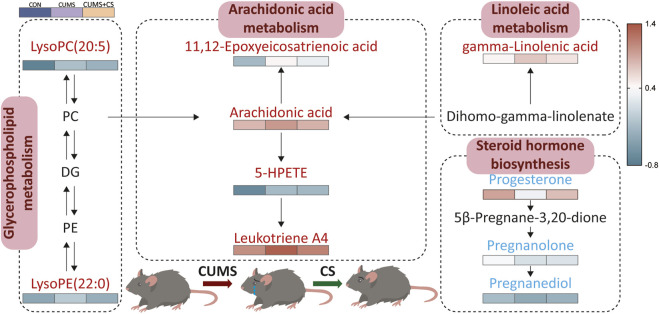
Metabolic pathway of mainly differential metabolites under effect of chronic unpredictable mild stress (CUMS) and *Cuscutae Semen* (CS). “CON” and “CUMS+CS” represented control group and CS treatment group, respectively. The color bar from blue to red represented low to high intensity after logarithmic transformation with a base of 10. Each group contained six samples, maintaining consistency across all groups.

Utilizing the quantitative and qualitative information of metabolites, metabolic pathway analysis was conducted through the KEGG platform. In summary, there are four pivotal pathways associated with altered metabolites (see Figure 6), which included arachidonic acid metabolism, glycerophospholipid metabolism, linoleic acid metabolism, and steroid hormone biosynthesis. Among these pathways, arachidonic acid metabolism and steroid hormone biosynthesis involved the most differential metabolites involved. Strikingly, the alternations in metabolites content within arachidonic acid metabolism and steroid hormone biosynthesis displayed reverse modes.

**FIGURE 7:**
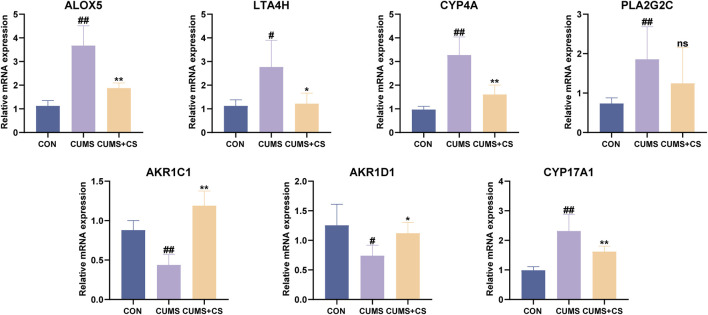
Effect of *Cuscutae Semen* (CS) on the expression of targets in the treatment of chronic unpredictable mild stress (CUMS) detected by real-time quantitative polymerase chain reaction (n = 6). “CON” and “CUMS+CS” represented control group and CS treatment group, respectively. Mann-Whitney *U* test was used to calculate significant difference. ^#^ *P* < 0.05, ^##^ *P* < 0.01, compared with CON group. ^*^ *P* < 0.05, ^**^ *P* < 0.01, compared with CUMS group. Each group contained six samples, maintaining consistency across all groups.

In order to further clarify the therapeutic mechanisms underlying CS treatment for CUMS, RT-qPCR techniques were employed to ascertain the expression levels of seven key targets associated with differential metabolites in the four metabolic pathways. As illustrated in Figure 7, the expression of ALOX5, LTA4H, CYP4A within the arachidonic acid metabolism was significantly upregulated after modeling. And there was a significant retraction of the expression following administration. Meanwhile, the CUMS group exhibited significantly reduced AKR1C1 and AKR1D1 expression and elevated CYP17A1 expression in steroid hormone biosynthesis compared to the CON group, while the mRNA levels of AKR1C1, AKR1D1 and CYP17A1 significantly changed in the opposite direction after CS pretreatment. Besides, the mRNA expression of PLA2G2C in the arachidonic acid metabolism and glycerophospholipid metabolism was significantly increased subsequent to model establishment.

## 4 Discussion

In this study, after inducing the depression-like model in female mice using the CUMS method, ovarian functionality was compromised. Behavioral assessments revealed that CS effectively alleviated depressive-like behavior in female mice. Furthermore, after the administration of CS, a noticeable restoration of the impaired ovarian functionality in the female mice was observed. Based on untargeted LC-MS-based metabolomics, 10 differential metabolites were found to be associated with the medication of CS on depression-induced ovarian dysfunction. Meanwhile, four metabolic pathways, including arachidonic acid metabolism, glycerophospholipid metabolism, linoleic acid metabolism and steroid hormone biosynthesis, were identified as potential therapeutic mechanisms underlying the treatment of ovarian dysfunction by CS. The expression levels of seven key targets associated with metabolites in the pathway were determined, further elucidating the potential mechanisms of CS treatment.

The increasingly utilized CUMS-induced modeling method was a valuable tool for investigating potential therapeutic interventions for ameliorating depression (Huang et al., 2023). The behavioral results clearly demonstrated that, following CUMS modeling, the mice showed a decrease in sucrose consumption, as well as an increase in both tail suspension time and swimming immobility. The reduced sucrose intake indicated a diminished pleasure response (Markov, 2022). Additionally, the prolonged tail suspension time and increased swimming immobility suggested heightened despair-like behavior (Li et al., 2023). Collectively, these behavioral changes indicated the onset of depression-like symptoms in the mice, consistent with previous studies (Tian T. et al., 2022; Zhong et al., 2024). However, with CS intervention, the mice’s performance in behavioral tests showed significant improvement, demonstrating that CS alleviates depressive-like behavior. Furthermore, our study substantiated that depression caused by CUMS adversely exerted an impact on the ovarian function of female mice. The research had revealed females exhibited a higher propensity for depression than males, giving rise to an increased array of complications (Sassarini, 2016). Within our findings, the observed variation in the secondary and atretic follicles signified that the development of follicles showed dysplasia. Moreover, AMH levels served as an indicator of ovarian reserve function (Helden and Weiskirchen, 2017). An elevated FSH and LH level, as well as a reduced E2 level were key biochemical markers of premature ovarian failure (Beck-Peccoz and Persani, 2006; Chen et al., 2019). Previous studies suggested that imbalances in FSH, E2, LH and AMH hormone levels exerted deleterious effect on ovarian function and even reproductive function (De Vos et al., 2010; Jiao et al., 2021). Our findings also indicated similar outcomes regarding changes in hormone levels, including a significant decrease in E2 and AMH, along with a significant increase of FSH and LH. Thus, it was plausible that depression induced by CUMS may adversely affect women’s reproductive function, in alignment with earlier reports (Slavich and Sacher, 2019). Following CS treatment, there was a significant tendency toward recovery in depression symptoms and hormone levels. Research has substantiated that the primary constituents of CS were flavonoids and phenolic compounds, such as kaempferol, hyperoside and chlorogenic acids (Liang et al., 2022; Shi Y. et al., 2022). It was worth noting that these compounds had been reported for their capacity to confer antidepressant benefits and mitigate reproductive dysfunction (Gong et al., 2017; Shah et al., 2023). Meanwhile, our finding indicated that mice had a marked enhancement in behavioral tests, an apparent resurgence in hormone assessments and the restoration of ovarian function. The estradiol treatment group demonstrated notable effects in alleviating depression-like behavior and restoring ovarian function. This may be attributed to its interaction with estrogen receptors, which influences the development of depression, aligning with previous studies (Furuta et al., 2013; Tian J. S. et al., 2022). Furthermore, as a type of estrogen, estradiol is the primary hormone regulating ovarian growth and physiological function (Xie et al., 2017). Although the efficacy of CS did not surpass that of E2 administration, it provided new insights into CS as a potential alternative therapeutic agent for alleviating depressive-like behavior and reproductive dysfunction in CUMS-exposed mice.

To our knowledge, this study constitutes the inaugural metabolomics investigation into the therapeutic effects of CS on depression-induced ovarian dysfunction. It was noteworthy that 10 metabolites with reversed trends after CS administration, containing steroids and steroid derivatives, fatty acyls and glycerophospholipids, were detected in the difference of CON-CUMS. These metabolites were mainly associated with four metabolic pathways, constituting a potential mechanism of drug action pathways. Among them, the largest number of differential metabolites was involved in arachidonic acid metabolism. Arachidonic acid metabolism played a pivotal role in cardiovascular diseases, carcinogenesis, and the genesis of numerous inflammatory maladies (Wang et al., 2021). In a mouse model of depression induced by a high-fat diet, the serum levels of arachidonic acid were also significantly elevated in mice exhibiting depressive behavior (Yu et al., 2023). In addition, arachidonic acid and its metabolites play a crucial role in the onset, progression, and metastasis of ovarian cancer (Xia et al., 2024). Our findings suggested that CS could actively alleviate the increase arachidonic acid metabolism caused by modeling. Additionally, we found several distinctive metabolites in steroid hormone biosynthesis, which was reported to be very important for proper ovarian function (Salilew-Wondim et al., 2015; Park et al., 2022). Among these compounds, progesterone played a crucial role in regulating anxiety in women, functioning as an anxiolytic steroid (Reddy et al., 2005). Additionally, pregnanediol and progesterone were key indicators for assessing ovarian function in females (Pepperell et al., 1975). It was worth noting that estrogen and progesterone also contributed to the pathophysiology of other neuropsychiatric disorders, such as obsessive-compulsive disorder, by enhancing 5-hydroxytryptamine signal transduction (Karpinski et al., 2017). In general, CS could alleviate depressive-like behavior and associated ovarian dysfunction by modulating these metabolites in mice.

Following the analysis of the metabolic pathways, seven genes including ALOX5, LTA4H, CYP4Y, PLA2G2C, AKR1C1, AKR1D1 and CYP17A1 were employed to elucidate further the potential mechanisms underlying CS treatment of depression. Among these, ALOX5, LTA4H, CYP4A and PLA2G2C were upstream or downstream gene targets of differential metabolites in arachidonic acid metabolism. These targets were reported to exhibit a positive correlation with the inflammation levels in tissues (Hui, 2012; Gomez et al., 2020; Liang et al., 2021; Huttl et al., 2023). Notably, PLA2G2C was also implicated as a target associated with metabolites in glycerophospholipid metabolism. In the present study, we discerned a significant increase in the expression of targets and relative contents in arachidonic acid metabolism subsequent to exposure to CUMS. It was noteworthy that inflammation assumes a pivotal factor in the manifestation of depression (Tao et al., 2021). It could be reasonably inferred that the inflammation induced by depression might play a certain contributory role in impairing ovarian function. In our findings, the correlated expression within arachidonic acid metabolism was significantly mitigated following the administration of CS, signifying the potential of CS to ameliorate tissue inflammation induced by CUMS. Thus, CS elicits antidepressant-like effects in female mice, which effects appear to be mediated by inhibition of inflammation via the arachidonic acid pathway.

Moreover, AKR1C1, AKR1D1, and CYP17A1 were identified as relevant targets of discriminant metabolites in steroid hormone biosynthesis. Fascinatingly, the content of differential metabolites in this pathway decreased in mice following CUMS modeling. There was evidence indicating that the decrease of AKR1C1 and AKR1D1 played an important role in the formation of ovarian tumor tissues and inflammation (Ji et al., 2005; Nikolaou et al., 2021). And previous studies suggested that an upregulation of CYP17A1 expression underscore pivotal roles in the pathogenesis of polycystic ovary syndrome (Kobayashi et al., 2020; Zeng et al., 2020). These supported the notion that the aberrations in steroid hormone biosynthesis might potentially underlie disturbances in hormone levels and impairments in ovarian functionality. Furthermore, it also advanced the proposition that CS constituted a favorable therapeutic intervention for CUMS and CUMS-induced ovarian complications. Interestingly, we discovered that AKR1C1, AKR1D1 and CYP17A1 belonging to the same pathway collectively governed the levels of progesterone. Collectively, our results further confirmed the potential regulatory mechanisms for CS administration in the CUMS model. We acknowledge several limitations in this study. First, while our results demonstrate the efficacy of CS, we did not investigate multiple dosing regimens or determine the optimal dose. Second, although CS exhibited positive effects in animal models, the CUMS model has certain limitations. Specifically, the CUMS model primarily assesses anhedonia and despair behaviors, but it does not fully capture the complex symptoms of human depression, such as cognitive impairments or suicidal tendencies (Hayley et al., 2021; Shi L. S. et al., 2022; Fountoulakis et al., 2023). Consequently, the CUMS model cannot entirely replicate the intricate pathogenesis of depression in humans. Additionally, there is a lack of clinical pharmacological evidence to substantiate CS’s potential activity in human subjects. Future research will focus on investigating the effects of CS through multiple-dose treatment experiments to establish the dose-response relationship. Additionally, we will conduct further research around CS, delve into its specific bioactive components, reveal its therapeutic mechanism, and confirm its potential activity through relevant clinical trials. Due to the interaction between E2 and FSH (Dewailly et al., 2016), a crucial focus of our future research will be to investigate the effects and underlying mechanisms of E2 intake on the regulation of FSH levels in the current model.

## 5 Conclusion

This study employed untargeted metabolomics to explore the therapeutic mechanisms of CS in alleviating depression-induced ovarian dysfunction in female mice. The findings demonstrate that CS effectively mitigates depressive-like behaviors and ameliorates reproductive dysfunction associated with CUMS. Key metabolic pathways implicated in the therapeutic effects of CS screened by metabolomics include arachidonic acid metabolism, glycerophospholipid metabolism, linoleic acid metabolism, and steroid hormone biosynthesis. Additionally, 10 pivotal metabolites and seven key molecular targets were identified, providing valuable insight into the regulatory effects of CS on these pathways. This research underscores the potential of CS as a medical food for addressing depression-induced ovarian dysfunction, offering a theoretical foundation for further exploration of its therapeutic applications in ovarian dysfunction.

## Data Availability

The original contributions presented in the study are included in the article/Supplementary Material, further inquiries can be directed to the corresponding authors.
