# Supplementary material for: Cuscutae Semen in depression-induced ovarian dysfunction: metabolomics with UPLC-QToF-MS in female mice
Source: Front Mol Biosci. 2025 Apr 30;12:1595602. doi: 10.3389/fmolb.2025.1595602 (PMC12074923; doi:10.3389/fmolb.2025.1595602)
Supplement: Supplementary file 1 [file Supplementaryfile1.docx]

***Supplementary Material***

***Cuscutae Semen* in Depression-Induced Ovarian Dysfunction: Metabolomics with UPLC-QToF-MS in Female Mice**

**Contents**

**Table S1** Chronic unpredictable stress protocol

**Table S2** The primer sequences for RT-qPCR

**Figure S1** The ethical review document

Table S1 Chronic unpredictable stress protocol

|  | The first week | The second week | The third week | The fourth week |
| --- | --- | --- | --- | --- |
| Mon. | Bondage 4h | Wet sawdust 12h | Food-depriving 12h | Reversed light/dark cycle |
| Tue. | Food-depriving 12h | Water-depriving 12h | Bondage 4h | Food-depriving 12h |
| Wed. | Wet sawdust 12h | Tail suspension 2h | Crowded housing 5h | Crowded housing 5h |
| Thur. | Empty cage 12h | Cold water swimming 5min | Noise interference 15min | Water-depriving 12h |
| Fri. | Water-depriving 12h | Bondage 4h | Wet sawdust 12h | Wet sawdust 12h |
| Sat. | Cage tilt 12h | Food-depriving 12h | Water-depriving 12h | Tail clamp 1min |
| Sun. | Cold water swimming 5min | Cage tilt 12h | Empty cage 12h | Cage tilt 12h |

Mice were subjected to a random exposure of a sequence of mild stressors (12 in total, 1 per day) over the course of 28 days. Bondage, put the mouse in a fixer. Wet sawdust, pour water into the mouse litter. Food-depriving, the mice were deprived of food. Reversed light/dark cycle, 12 hours upside down day and night. Water-depriving, the mice were deprived of water. Tail suspension, the mouse tails are fixed and suspended. Crowded housing, the density of mice in the cage was significantly increased. Empty cage, feed the mice in bedding-free cages. Cold water swimming, the mice were forced to swim in ice water for 5 minutes. Noise interference, mice were exposed to high-intensity white noise environment. Cage tilt, tilt the cages of mice. Tail clamp, clamp the tail root of the mouse with a soft clip.

Table S2 The primer sequences for RT-qPCR

| **Gene** | **Primer** | **Sequence (5'-3')** | **PCR Products** |
| --- | --- | --- | --- |
| Mus GAPDH | Forward | GAGAGTGTTTCCTCGTCCCGTA | 252bp |
|  | Reverse | CCTCACCCCATTTGATGTTAGT |  |
| Mus ALOX5 | Forward | GCTTATCTGCGAGTATGGC | 158bp |
|  | Reverse | GGATGTCTTCCGTGCTGT |  |
| Mus LTA4H | Forward | GCTTCAGAAGATGGTTACGGC | 130bp |
|  | Reverse | AAGGCAGCGAGATCCTTGAAT |  |
| Mus CYP17A1 | Forward | TCAGGGATGACCAGAAACT | 111bp |
|  | Reverse | TGAAGATGAGCGTAGACAGAT |  |
| Mus AKR1D1 | Forward | TGGAGTGCCACCCGTATT | 222bp |
|  | Reverse | TCCCTCGCTGGATGTTGA |  |
| Mus PLA2G2C | Forward | AGCAGCTCTTCCCGACCA | 123bp |
|  | Reverse | CCCAGACAGCAAAGCTCTTCA |  |
| Mus CYP4A | Forward | CCAAAATCCAAGGCCTGAACAT | 105bp |
|  | Reverse | GACTATGCAGGGTAGATTTAGATGA |  |
| Mus AKR1C1 | Forward | CCATCCGAAGCAAGATAGCA | 113bp |
|  | Reverse | CTGTTCCAAGCAGACCCGTA |  |


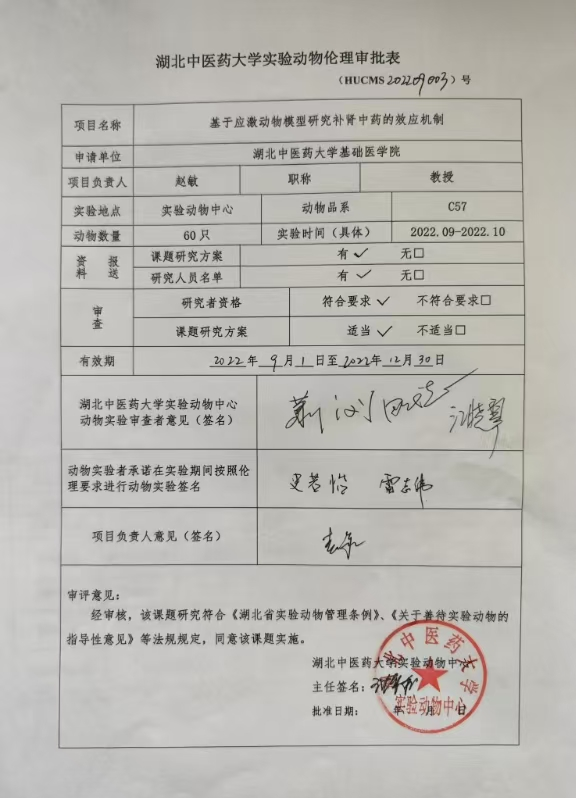


Figure S1 The ethical review document
